# “I Just Want to Feel Safe Going to a Doctor”: Experiences of Female Patients with Chronic Conditions in Australia

**DOI:** 10.1089/whr.2022.0052

**Published:** 2022-12-22

**Authors:** Lea Merone, Komla Tsey, Darren Russell, Cate Nagle

**Affiliations:** ^1^ College of Healthcare Sciences, James Cook University, Townsville, Queensland, Australia.; ^2^ College of Arts, Society, and Education, James Cook University, Cairns, Queensland, Australia.; ^3^ Cairns and Hinterland Hospital and Health Service, Queensland Health, Cairns, Queensland, Australia.; ^4^ Townsville Hospital and Health Service, Townsville, Queensland, Australia.

**Keywords:** health, chronic conditions, experiences, feminism, women's health

## Abstract

**Background:**

The androcentric history of medicine and medical research has led to an ongoing sex and gender gap in health research and education. Sex and gender gaps in research and education may translate into real-life health inequities for women. This study aimed to explore the experiences of female patients with chronic health conditions in the Australian health system, considering existing sex and gender gaps in medicine.

**Methods:**

This qualitative study used semistructured in-depth interviews with a sample of adult women with chronic conditions in Australia. Thematic analysis was undertaken, guided by Braun and Clarke. Software NVivoX64 assisted in the management of the data. Coding was performed before grouping into subthemes and central themes. To allow for potential researcher biases, the principal researcher engaged in the practice of reflexivity, including the writing of detailed notes during analysis.

**Results:**

Twenty adult Australian women with chronic conditions were interviewed. Diagnoses were varied and included Ehlers–Danlos syndrome, chronic fatigue syndrome, functional neurological disorder, and inflammatory bowel disease. Four central themes emerged: diagnostic difficulties; spectrum of health care experiences; understanding medical complexity; and coping with symptoms.

**Conclusions:**

Women with chronic conditions in Australia report pain, fatigue, and suffering that significantly impacts upon their daily lives. There was a shared experience of feeling that the pain and suffering of women was dismissed or not taken seriously. Many women expressed trauma because of their experiences in health care and often this led to a fear of accessing health services. The participants highlighted a need for more knowledge, understanding, and empathy from health care practitioners.

## Background

Medical research has historically been androcentric^1,2^; most research has been performed on the bodies of the average male and generalized to females.^3–5^ There is evidence to suggest that globally, this sex and gender gap in health research is ongoing^6^ and a recent cross-sectional analysis highlighted a specialty-based sex and gender gap in Australian health research.^7^ This research gap is thought to possibly translate into a gap in medical education whereby medical schools and clinical textbooks frequently omit women's health sections outside of reproductive and sexual health.^8^

It is possible that sex and gender gaps in research and education translate into real-life health inequities for women. The paucity of research of females may result in the greater prevalence of medically unexplained symptoms (MUS) observed in women,^9^ possibly as a result of a lack of knowledge surrounding the female presentation of disease and response to treatment. Research has demonstrated a gender bias in the diagnosis of MUS and somatic symptom disorder, with female patients significantly more likely to be diagnosed with these syndromes than male patients.^10^ The specialty with the highest prevalence of MUS, determined in a U.K. epidemiological study, was gynecology.^9^

Women wait longer for a diagnosis than men.^11^ In the emergency department women also wait longer for analgesia and have their pain inadequately managed compared with men.^11^ Gender discrepancies in time to diagnosis are observed in cancers; in the diagnosis of bladder cancer women with hematuria experience longer waiting times for urology assessment than men^12^ and similar delays have also been observed in the diagnosis of colorectal, gastric, head and neck, lung, and lymphoma cancers.^13^

A recent survey of women with chronic conditions in Australia demonstrated women wait an average of 4 years for a definitive diagnosis and almost half of these women are rediagnosed at least once.^14^ This survey ascertained that of the women who were rediagnosed, 32% were originally given a diagnosis of a psychological or MUS and later rediagnosed with an organic condition.^14^ Women are notably more likely than men to be dismissed or told their symptoms are “in their head” before a diagnosis of organic disease is made.^15^ Despite noting gaps in women's research and health care, little research has been conducted to capture the experiences of women with chronic conditions in the Australian health care system.

This semistructured in-depth interview study aimed to explore the potential effects of the research gap in medicine on the experiences of female patients with complex chronic health conditions in the Australian health system.

## Methods

### Research team

The design, analysis, and writing team consisted of four members; three professors, and one PhD candidate, two identify as women and two as men. The principal researcher, who also coded the data, identifies as a white, disabled woman. This study was guided by feminist social constructivism, using an inductive approach to analysis.

### Participants

Adult women with at least one chronic condition and residing in Australia since birth or early childhood were eligible to participate. Regrettably, women unable to speak or understand spoken English were excluded as this study was unfunded. We recruited and interviewed women until thematic saturation^16^ was reached and then continued interviews for two further participants. Women were recruited *via* social media. As per a recent survey analysis,^14^ the study was advertised using relevant hashtags and notices in appropriate Australian chronic conditions support groups, including: chronic pain; endometriosis; lupus; fibromyalgia; general chronic disease; irritable bowel syndrome (IBS); autoimmune disease; heart disease; depression; anxiety; Ehlers–Danlos syndrome (EDS); cancer; asthma; diabetes; postural orthostatic tachycardia syndrome (POTS), and many more. Interested participants were required to voluntarily contact the principal researcher and following provision of the information material and informed consent, were recruited to the study.

### Procedures

The interviews were conducted using a mixture of videoconference and telephone formats. The data collection tool was devised using guidelines from “Learning From Strangers, the Art and Method of Qualitative Interview Studies,”^17^ “Feminist Research in Theory and Practice,” “Feminist Research in Practice,” and “Grounded Theory Research: a Design Framework for Novice Researchers”^18^ and informed by a review of the literature. The principal researcher conducted semistructured interviews, asking women to tell the story of their condition and experiences in the health care system in Australia. If topics were not raised by the participant, the interviewer prompted responses using the questions outlined in Table 1. Demographic characteristics collected were: age; state of residence; primary diagnosis; and secondary diagnoses. Primary diagnosis was determined by the participant as either their first diagnosis, or the diagnosis which is most prominent in their life.

**Table 1.** Outline of Questions Used to Prompt Responses From Participants

1. How does your diagnosis/diagnoses affect you on a day-to-day basis?
2. Talk me through the journey of your condition. Perhaps start with your symptoms and diagnosis through to now.
3. Were you previously given another diagnosis for this condition? If so, tell me more about it.
4. How have health professionals responded to your illness?
5. Tell me about any positive experiences you've had in health care during your journey.
6. What would you like to see change or improve with treatment regarding your condition?

With informed consent, interviews were audiorecorded on a digital voice recorder and participants were informed that they could withdraw at any time without explanation up to the point of analysis. Where the interviewer noticed any distress, interviews were paused, and participants given the opportunity to cease the interview and either resume another day or withdraw entirely. A professional transcription service was used to transcribe interviews. There was no identifying information provided to the transcription service and each participant was identified only with a unique identifying code, which comprised the interview number and primary diagnosis. This study was approved by the University Human Research Ethics Committee (H8547).

### Data analysis

Inductive thematic analysis was guided by the framework described by Braun and Clarke^19^: familiarization with the data; transcription of verbal data; generation of initial codes; searching for themes; reviewing themes; defining themes; and producing a report. The principal researcher read the transcripts several times, coding iteratively. When no new themes emerged and thematic saturation was reached, interviews continued for two more, following which a full analysis was conducted.

To mitigate the risk of potential researcher biases and opinions affecting the results of this study, the principal researcher engaged in the practice of reflexivity according to the model developed by Alvesson and Skoldberg (2009).^20^ The principal researcher kept a series of reflective notes following each interview regarding emotional responses and thoughts that occurred at the time. These notes were used when coding and theming the data to reduce the impact of the experiences of the researcher on how the data were interpreted. Trustworthiness and rigor of the study were enhanced by monitoring the research process reflexively. To strengthen credibility, ensure language was free from bias and demonstrate respect to the chronic condition community, this article was reviewed by two external female consumer stakeholders with chronic conditions.

## Results

Twenty adult Australian women with chronic conditions were interviewed throughout January and February 2022 for a median of 39 minutes (IQR 22.25, range 17–91). The age range of participants was 21–67 years (median 42, IQR 19.25). The demographics of the participants are provided in Table 2.

**Table 2.** Participant State/Territory of Residence and Primary Diagnoses

Demographic	Number of participants
State/territory of residence	
Victoria	7
Queensland	5
New South Wales	2
South Australia	1
Northern Territory	1
Australian Capital Territory	1
Tasmania	1
Not stated	2
Primary diagnosis	
EDS	8
ME/CFS	3
Chronic pain syndromes	2
Depression	1
Ulcerative colitis	1
Irritable bowel syndrome	1
FND	1
Pelvic congestion	1
Long-term sequelae of encephalitis	1

EDS, Ehlers–Danlos syndrome; FND, functional neurological disorder; ME/CFS, myalgic encephalomyelitis/chronic fatigue syndrome.

Nineteen out of the 20 women had one or more secondary diagnosis. Secondary diagnoses included: POTS; depression; complex posttraumatic stress disorder (PTSD); endometriosis; dysautonomia; supraventricular tachycardia; and fibromyalgia.

Participant responses were analytically coded into four main themes (Table 3): diagnostic difficulties; spectrum of health care experiences; understanding medical complexity; and coping with symptoms.

**Table 3.** Three Levels of Themes in This Study

Theme	Subthemes	Sub—subthemes
Diagnostic difficulties	Challenges in obtaining a firm diagnosis	Delayed diagnosis Benefits of a diagnostic label Access to clinicians Seeking another opinion Own responsibility to solve the problem Consumer stakeholders Assumptions of doctors Impact of COVID-19
	Misdiagnosis	Psychological diagnosis Being too young
	Unclear diagnosis	Lack of objective evidence of disease Multiple diagnoses Normal test results Nonspecific test results
Spectrum of health care experiences	Dismissed	Disbelieved Abandoned by professionals Not being listened to
	Sexism/misogyny	Experiences of female friends/other patients Need for culture change in medicine
	Trauma and safety	Denied help Stigma Sense of blame Rude health care staff Bullying and abuse from health care staff Accused of faking symptoms Fear of accessing services Weight stigma Vulnerability Shame and humiliation Low expectations for help Gaslighting Insensitivity Frustration with doctors
	Being listened to	Empathy Being listened to Being taken seriously Doctors admitting they do not know
	Advocacy	Self-advocacy Family advocacy Doctors as advocates Doing own research
Understanding medical complexity	Need for more knowledge and understanding	Need for more education for clinicians Need for staff to understand women's health Consulting consumer stakeholders Problems with research and funding Learning from past errors Desire for partnership with health care staff
	Medical complexity	Problems commencing in childhood Multiple diagnoses Flexible or adaptive practice
	Altruism to tell their story	Appreciation for this research Importance of telling their story
Coping with chronic conditions and health care experiences	Suffering with symptoms	Pain Fatigue Cognitive dysfunction Variability of symptoms Self-management of symptoms Improvement with treatment
	Psychological and social impact of chronic illness	Sense of failure Anxiety and depression Social isolation Loss of identity Support from peers Support from social media Patients as communities Sense of inconvenience to carers Acceptance Strength and persistence Futility Hope
	Poorer quality of life	Help from NDIS Disability Burden

### Diagnostic difficulties

Participants discussed their journey from initial symptoms to diagnosis and a significant theme that was expressed by almost all women was the difficulty in obtaining a firm diagnosis. Recurrent misdiagnosis and rediagnosis were common, and several women consequentially had difficulty identifying their primary diagnosis.

“I think the diagnosis I don't have is my most important diagnosis, but I can't tell you what that is and I can't tell you which of the things going wrong with me is most important today” (Interviewee 20, diagnosis of EDS with POTS and lupus).

“But finding the diagnosis, no one was interested.” (Interviewee 10, diagnosis of EDS)

Participants frequently attributed their difficulties to challenges convincing doctors and health care staff that they felt unwell and had a medical condition.

“It can be really difficult trying to convince doctors that you are sick” (Interviewee 10, diagnosis of EDS).

“It's really really hard then to go back on 20 years of medical records saying that there's nothing really wrong with you and you're basically making it up” (Interviewee 13, diagnosis of EDS).

As part of the diagnostic process, it was common for the women interviewed to have been given a mental health diagnosis before being rediagnosed with a physical condition. A psychological misdiagnosis was associated with even greater difficulties obtaining a diagnosis for physical symptoms.

“They were like, you're just anxious, it's just anxiety. I'm like, what that explains my liver function?” (Interviewee 13, diagnosis of EDS).

“Everything that happens once you get the psyche label attached to you is they forget everything else, and it is Hell.” (Interviewee 19, diagnosis of FND).

In some cases, the participants expressed that owing to the difficulties they experienced in obtaining a clear diagnosis, they had ultimately stopped seeking answers for their symptoms.

“I gave up looking for formal diagnoses” (Interviewee 08, diagnosis of ME/CFS).

Many women emphasized the importance of obtaining a diagnostic label for their mental and physical wellbeing. Diagnostic labels were associated with having a condition that can be treated and is taken seriously by doctors. Additionally, a diagnostic label was noted to be important to be able to communicate their problems to other health professionals.

“When I received a diagnosis of EDS it changed—a lot of things did change because the doctors were able to recognize that this is actually what's going on and it wasn't this huge medical mystery where the patient is just making things up in their head” (Interviewee 02, diagnosis of EDS and POTS).

“I somehow have to convey that I have all the symptoms that would qualify me for a diagnosis of lupus, which is very, very unhelpful for my relationship with every new doctor I have to explain this to….. I wish I had said to him, but do you understand the impact it has on my relationship with every other doctor, that I have lupus that we're not calling lupus?” (Interviewee 20, diagnosis of EDS with POTS and lupus).

Many of the women who experienced misdiagnosis or received an unclear diagnosis, expressed frustration that doctors often felt them “too young” to be experiencing their symptoms and believed that this posed a significant barrier to a firm diagnosis and treatment.

“Saying, because of my age I must be bulimic” (Interviewee 12, diagnosis of dysautonomia).

In response to unclear diagnosis, misdiagnosis, or lack of effective symptom relief, several women reported accessing alternative health care services, such as Integrative General Practitioners; Chinese and herbal medicine practitioners; and homeopathic professionals. Diagnosis and treatment from alternative practitioners were associated with a greater sense of wellbeing, feeling believed, and increased patient satisfaction, even if physical symptoms did not always resolve.

“She stood me up, sat me down, took my blood pressure and heart rate, stood me up and did it again and went no, no, no, this is not okay, a 19-year-old should not be doing this, you go back to the GP and ask for a cardiologist. So, my homeopath triggered my cardiology referral” (Interviewee 20, diagnosis of EDS with POTS and lupus).

### Spectrum of health care experiences

**FIG. 1.**
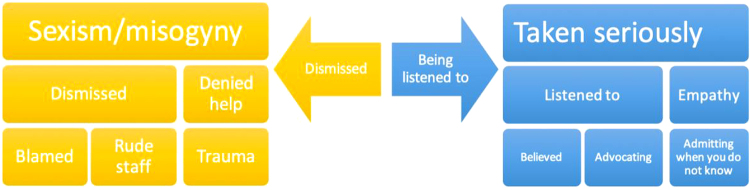
Experiences as a spectrum during encounters with health care staff in Australia.

There was a wide variety of experiences in the health care system that were distributed along a spectrum. At one end of the spectrum, many women felt dismissed by their doctors, with several expressing they felt this was due to sexism. Those who felt dismissed reported being denied help with their illness, and there was a sense of blame for not responding to treatments or for being unwell. Some of the women reported that health care staff were rude to them and accused them of faking their illness. At the other end of the spectrum, positive experiences in health care were associated with being listened to, being believed, and other doctors advocating for their treatment. The spectrum of experiences is highlighted in Figure 1.

All the women interviewed expressed feeling dismissed on at least one occasion. This was associated with adverse emotions, such as feeling frustrated with their doctors or feeling vulnerable.

“When I saw the pain specialist, he was very dismissive of my pain” (Interviewee 11, diagnosis of EDS).

“It's also very frustrating because it's taken ten years for someone to notice” Interviewee 10, diagnosis EDS).

“It's frustrating and I've definitely been brought to tears by it on a number of occasions because you're vulnerable” (Interviewee 01, diagnosis of chronic pain and CIN 3).

Being dismissed was not only frustrating, but also strongly related to a feeling that women were not believed, or that the symptoms were entirely psychological.

“Honestly, I will let something go until I'm near being hospitalized now because of a lifetime of being dismissed or not believed” (Interviewee 01, diagnosis of chronic pain and CIN 3).

“It's the first line of well, we couldn't figure it out easily, so maybe you're just anxious” (Interviewee 20, diagnosis of EDS with POTS and lupus).

“Lots of people since have said it's in my head, but the ones I listen to say it's not. Off to the psychologist. They did nothing.” (Interviewee 14, diagnosis of diagnosis of chronic pain).

Feeling as though they were not receiving the help they needed for their condition was common. This was accompanied by a sense of abandonment and having to learn to manage symptoms for themselves, with inadequate symptom management and follow-up.

“I just remember I didn't get any help during that period, I just had to tough it out, basically” (Interviewee 02, diagnosis of EDS and POTS).

“They sent me home from hospital while I was still paralyzed, basically said it's not a stroke, get used to it” (Interviewee 10, diagnosis of EDS).

“They weren't giving anything for the pain” (Interviewee 13, diagnosis of EDS).

“I would say he's been supportive in that very general sense, but there's never been a huge amount of follow-up or anything” (Interviewee 04, diagnosis of depression with PTSD).

Alongside frustration at diagnostic difficulties and being dismissed, there was a strong sense of trauma, including stigma, prejudice, shame, and blame received from health services from many of the women who were interviewed.

“The way in which you're treated in emergency department is like you are just drug seeking. I've certainly felt like I'm also doctor shopping at times” (Interviewee 07, diagnosis of EDS with endometriosis).

“I feel deeply ashamed and a great amount of guilt about my medical situation” (Interviewee 02, diagnosis of EDS and POTS).

Several women attributed their adverse experiences and difficulties in obtaining a diagnosis and adequate treatment to systemic and institutionalized sexism and misogyny.

“The GP was just pretty shocking. She just kind of told me to suck it up and that I should be a better wife to my husband” (Interviewee 04, diagnosis of depression with PTSD).

“I think this is just more of the generalized socialization of women, that it's all in our heads…. there's a lot of misogyny within the way that women's pain is assessed and interpreted” (Interviewee 11, diagnosis of EDS)

“I spent a lot of years being dismissed as an anxious woman rather than having my physical symptoms investigated” (Interviewee 13, diagnosis of EDS).

Additionally, one woman reported receiving an out-of-date and widely accepted misogynistic diagnosis.

“My discharge summary says hysteria is my diagnosis. Imagine how that feels to read that the health service thinks you have hysteria?” (Interviewee 19, diagnosis of FND).

Furthermore, there was a sense that each doctor was limited to knowledge within their own specialty, and this was a hinderance to diagnosis as doctors would be unable to consider conditions outside of their own expertise. This was accompanied by a desire for health care to be more holistic.

“I've just felt that every doctor is just ticking off their one box and quite dismissive of how it's impacting my whole life” (Interviewee 11, diagnosis of EDS).

The reported adverse experiences and sense of being disbelieved were linked to use of the words “trauma,” “gaslighting,” and an associated fear of accessing health services.

“The gaslighting is implicit, even if somebody's telling you they believe you that you're not doing okay, they're saying, well, everything is ok. Your tests came back okay, so you must be okay” (Interviewee 06, diagnosis of ME/CFS).

“I believe I have been pushed to a number of psychological crises due to the experiences with (the medical) professionals that I have explained” (Interviewee 02, diagnosis of EDS and POTS).

“It was enough to turn me off getting anymore treatment from medical providers for these mental health issues for several—I guess five or six—years” (Interviewee 04, diagnosis of depression with PTSD).

Almost all the participants described traumatic adverse events in their experience of health care, including bullying and intimidating behavior from health care staff and some were overtly accused of faking their illness.

“I then heard the paramedic next to me telling me to stop it (name omitted) I know you are faking this; I know you have pseudo-seizures” (Interviewee 02, diagnosis of EDS and POTS)

“They were accusing me of being bulimic, they were accusing me of purposely doing something, purposely taking I think they're called loop diuretics or something” (Interviewee 12, diagnosis of dysautonomia).

Adverse experiences were associated with a fear of accessing services and a sense from some women of being unsafe in hospital owing to professionals misunderstanding their complex conditions.

“There's a ton of fear every time you step into the room, because you're worried about what the reaction to your new issues are or your old issues, which I'm sick of hearing about.” (Interviewee 07, diagnosis of EDS with endometriosis).

“We have literally stopped calling 000 because I am at more risk of harm in a hospital from people who don't follow basic seizure protocols” (Interviewee 19, diagnosis of FND).

“I just want, I don't know, to feel safe going to a doctor. I really am petrified of doctors” (Interviewee 15, diagnosis of pelvic congestion).

At the other end of the spectrum, positive experiences in health care were strongly related to finding a doctor who listened and took their illnesses seriously. Doctors being able to admit when they did not have an answer was also something valued by several participants.

“She actually said to me, I'm happy to do some reading about this because I don't understand. I nearly fell off my freaking chair!” (Interviewee 07, diagnosis of EDS with endometriosis).

“I saw a doctor there who was the first doctor who actually listened to me” (Interviewee 14, diagnosis of chronic pain).

Empathy was a high-rated quality in a health care professional and associated with positive feelings toward health care.

“When you're already vulnerable and terrified you lose your voice and you're really reliant on healthcare professionals to use their empathy, foresight and advocate for you and care for you” (Interviewee 01, diagnosis of chronic pain and CIN 3).

Advocacy in all forms was another strong theme surrounding experiences in health care, with women often describing the importance of self-advocacy, advocacy from family members, and sometimes advocacy from doctors, particularly general practitioners.

“I'm quite, I think, a confident, vocal advocate for my needs, but ironically that's quite difficult when you have a neurological condition in and of itself.” (Interviewee 18, diagnosis of long-term sequelae post-encephalitis).

“Friends and family know that I've got a very high pain threshold. If anything they're the ones screaming by my side advocating for me if they're there at the time.” (Interviewee 01, diagnosis of chronic pain and CIN 3).

“About a year into it my GP said, look, this is ridiculous. I'm just going to write to the hematology department and ask them to treat you” (Interviewee 20, diagnosis of EDS with POTS and lupus).

### Understanding medical complexity

Participants almost unanimously identified that there was a need for more knowledge, research, and education for clinicians to understand complex chronic conditions that affect primarily women, such as EDS and myalgic encephalomyelitis/chronic fatigue syndrome (ME/CFS).

“I think a lot of education needs to be done from the first point of call for people because you just get blank stares or you get disbelief, so you get gaslit and it's horrible.” (Interviewee 09, diagnosis of ME/CFS).

“Education all round. Treatment research. There's no money going into pain research. It's not even a recognized disease.” (Interviewee 14, diagnosis of chronic pain).

“I think there's just seriously still a dearth of understanding and literacy about mental health issues even amongst some professionals, which is pretty poor I think given a country like Australia especially—I think our health system should be doing much better than that.” (Interviewee 04, diagnosis of depression with PTSD).

The understanding and knowledge of medical complexity and chronic conditions of frontline doctors such as ED physicians and general practitioners was noted to be an area for development.

“There's a huge problem with the way doctors diagnose. When you go to a GP these days, if you've got your average run-of-the-mill stuff, they can cope with that.” (Interviewee 13, diagnosis of EDS).

“Especially our frontline needs a lot more training.” (Interviewee 01, diagnosis of chronic pain and CIN 3).

There was an expressed need for doctors and other health care professionals to be more empathetic and understanding and less dismissive of female patients with complex and chronic conditions. This theme was particularly common among women who reported being actively engaged in patient advocacy.

“I just want people to be less judgmental, more empathetic and compassionate with every sort of disease” (Interviewee 07, diagnosis of EDS with endometriosis).

The majority of women identified that their problems began in childhood and were often rediagnosed later on as adults as their medical complexity increased. Many women expressed that they have very complex medical histories and needs, with multiple diagnoses and adverse reactions to treatments. Additionally, many felt that the medical system was not equipped to manage chronic and complex conditions and that anyone who did not fit the known diagnostic criteria is often dismissed.

“I've had such rare conditions that people have just kind of gone well, she must be making it up, she can't really have all these random symptoms that make nothing.” (Interviewee 10, diagnosis EDS).

“The concrete diagnosis that I have at the moment is POTS and EDS, but there are still ongoing things that could be diagnoses as well, like mast cell activation syndrome and gastroparesis but I've had a bit of trouble getting concrete diagnoses for those.” (Interviewee 02, diagnosis of EDS and POTS)

Many of the women found their experiences in the health services extremely distressing to discuss, however, there was a true sense of altruism to tell their story, with many women expressing appreciation for the opportunity to tell health professionals about their encounters.

“But it's important for people to know about this—that what happens to us in hospital, what happens with the ambulance calls and everything else that happens.” (Interviewee 19, diagnosis of FND).

“I want someone to hear this story, because I don't think—I don't believe it's individual doctors.” (Interviewee 20, diagnosis of EDS with POTS and lupus).

### Coping with complex chronic conditions and health care experiences

The most experienced symptom described by the women interviewed was pain, with almost all women reporting significant daily pain, most commonly of the joints or the abdomen. Several women used adjectives such as “excruciating,” “severe,” and “chronic” to describe their pain. Many women described reliance on analgesia and nerve blocks to manage their pain, yet despite requirements for pain relief, there was a sense of judgment from health care providers that impacted upon access to care.

“This GP was quite judgmental of this chronic pain issue, and I would say, in a sense, coaxed me to have a trial without any medications” (Interviewee 07, diagnosis EDS with endometriosis).

Following this, many women reported fatigue as a common but poorly understood symptom and a significant daily grievance,

“The biggest symptom at the moment for me is the fatigue” (Interviewee 10, diagnosis of EDS).

“Doctors do not understand – and I'm sorry for the generalization but just do not understand fatigue” (Interviewee 18, diagnosis of long-term sequelae of encephalitis).

Additionally, there was an overall sense of great suffering that severely impacted upon the daily lives of all the women who were interviewed.

“It doesn't give me one second break. It's equivalent to torturing someone” (Interviewee 19, diagnosis FND).

“Essentially, it's derailed most of my life” (Interviewee 06, diagnosis ME/CFS).

Owing to their daily symptoms and suffering, the women interviewed described the poorer quality of life they experience. Many of the women reported their capacity to work or perform general activities of daily life were affected.

“I don't really feel like I can live day to day like most people” (Interviewee 09, diagnosis of ME/CFS).

“If I'm able to make myself food, then I feel like that's a good day” (Interviewee 10, diagnosis of EDS).

“I managed to keep working for quite a while, but it was at the expense of a social life” (Interviewee 08, diagnosis of ME/CFS).

A few women reported feeling socially isolated and a loss of identity because of their symptoms and chronic conditions.

“I didn't have contact with anyone outside of my mum, so I was very socially isolated” (Interviewee 04, diagnosis of depression with PTSD).

“It certainly has really affected my identity, my self-identity” (Interviewee 18, diagnosis of long-term sequelae post-encephalitis).

Conversely, some women noted the importance of the patient community *via* support forums and social media.

“So, I'm connected in a group, an FND support group, and we meet once a month” (Interviewee 19, diagnosis of FND).

One participant, however, noted that the negative experiences of friends and those on support groups can impact on their own perceptions of the health system and increase anxiety around seeking health care.

“Because of these negative experiences that my friends have had with further investigations, I'm almost depleted” (Interviewee 16, diagnosis of IBS).

Similar to this experience, many women stated also suffering anxiety and depression, not as a cause of their chronic condition, rather because of their ongoing suffering, both physically and because of their own adverse experiences in the health care system.

“The anxiety in the past year has been crazy and it's not about my illness, I've always been ill…. But dealing with the professionals—you can't write your own referrals; you can't write your own pathology scripts to go and get a blood test. There are certain things you need a doctor for, and I can't get anyone to listen to me, it's just crazy” (Interviewee 15, diagnosis of pelvic congestion).

While all the women described physical and emotional suffering, there was also a wide sense of acceptance of their situation, often based in perceptions of doctors being unable or unwilling to offer further help.

“Just kind of accepted that this is life.” (Interviewee 10, diagnosis of EDS).

“Often, I will get ignored about it anyway if I ask, if I'm having a particularly bad flare-up from time to time, because it does flare up from time to time. It's very hard to convince anyone to investigate it further anyway. So, you just learn to deal with it yourself.” (Interviewee 01, diagnosis of chronic pain and CIN 3).

While there was great suffering reported, it must also be acknowledged that there was a strong sense of strength, with several women discussing how they engage in patient advocacy and “push through” their symptoms.

## Discussion

This qualitative interview study revealed that women with complex chronic conditions in Australia experience difficulties in obtaining a diagnosis and subsequently have experienced a spectrum of encounters with health care staff, ranging from feeling dismissed to feeling believed and listened to. Many women feel there is a poor understanding of medical complexity and women's health from health care staff. Furthermore, women suffer a wide range of symptoms that significantly impact upon their quality of life and activities of daily living.

The women in this study reported significant challenges and obstacles to obtaining a diagnosis for their conditions. Although there are many studies pertaining to female suffering and experience of chronic conditions,^21,22^ there is little literature surrounding the experiences of women with complex chronic conditions in obtaining a diagnosis and receiving medical treatment. All the women interviewed in this study described some degree of diagnostic difficulty with at least one of their chronic conditions and these difficulties were associated with a sense of frustration, feeling dismissed, or suspecting they were not believed or taken seriously. Researchers and clinicians have long noted that diagnostic difficulty and ambiguity lead to frustrations from clinicians^23^ and a sense of delegitimization or dismissal from patients,^24^ which may in turn result in mistrust of health care providers.^25^ Despite this, there is a paucity of literature comparing the diagnostic experiences of females and males and examining diagnostic journeys of female patients.

Many women described being misdiagnosed or rediagnosed on at least one occasion, this appeared more common where problems began in childhood and original diagnoses were later rediagnosed as more disease features manifested. This finding was supported by a recent (2022) survey of Australian women with chronic conditions, wherein almost half of the women reported being rediagnosed on at least one occasion.^14^ Misdiagnosis and rediagnosis may contribute to longer waiting times for an ultimate diagnosis, delays in treatment, and progression of the chronic condition.^14^

Many of the women interviewed stated their symptoms had at some point been attributed to psychological conditions, most commonly anxiety. Research has demonstrated that women are often diagnosed with a psychological or MUS condition before later being rediagnosed as having an organic condition.^14^ Furthermore, women are noted to be at greater risk than men of having their physical symptoms attributed to psychological causes, often without thorough investigation.^10^ Finally, prior research supports this study; women (and men) with chronic conditions often feel their anxiety is related to having a chronic condition, rather than anxiety causing their physical symptoms.^26^

Many of the participants reported significant and traumatic adverse experiences in the health care system, from being accused of faking their symptoms to being denied care and help. These experiences were associated with a sense of medical trauma and being “gaslit.” Medical gaslighting is a term that is becoming increasingly common in the literature and is defined as an experience of invalidation, dismissal, and inadequate medical care.^27^ Emerging research is demonstrating that medical gaslighting is felt particularly among female patients suffering with medically ambiguous or unexplained syndromes, such as ME/CFS and POTS.^28^

Several of the women in this study described experiencing trauma because of their health care experiences, rather than as a direct result of their symptoms. It is long noted that women are more likely to suffer from MUS or ambiguous, complex chronic illness than men, and that women suffering with these conditions must learn to manage them within the context of a hostile medical and social culture.^25^ In keeping with our findings, a 2015 focus-group study of Australian women with chronic conditions discovered that women have a sense of disempowerment and dismissal through interactions with health care systems.^29^ There was little literature from the patient perspective to support our findings of doctors accusing female patients of faking their illness or medical trauma and gaslighting, however, this may be owing to little recognition of these factors impacting upon patient care and a research oversight to date.

The work of Young et al, however, supports the idea that clinicians may view women as attention seeking, exaggerating or “mad” when presenting with complex, painful, and poorly understood conditions such as endometriosis.^30^ Other research has supported this gender bias in the care of chronic pain and diagnosis of chronic and acute conditions.^11,31–35^

Some of the women expressed they felt their traumatic experiences were due to systemic sexism. It is possible that adverse experiences in health care for women are related to an underlying misogynistic culture in medicine^29,30^ and medical research,^36^ lack of research knowledge,^37^ lack of education about women's health,^8^ and compassion fatigue from clinicians.^38^ A 2013 Swedish survey of adverse hospital encounters demonstrated that a feeling of “being wronged” in female and male patients was associated with not being believed, not being listened to, and feeling disrespected.^39^ Within this survey and the findings of an earlier (2007) study, women were noted to be overrepresented in terms of negative encounters.^39,40^

Despite a high number of reported adverse experiences in health care, every woman interviewed was able to describe at least one positive experience. Positive experiences were primarily associated with a feeling of being listened to and taken seriously and were often discussed with a tone of surprise and adverbs such as “actually.” This finding was supported by Wessel et al in their 2013 survey, noting being listened to, being treated with respect, and believing the patient were all consistent with positive encounters.^39^ The patient experience is known to affect outcome, health, and wellbeing, with positive interactions associated with better outcomes.^41^

Many women believed their adverse events to be related to a lack of knowledge and understanding about complex chronic conditions on the behalf of the health care providers. Research has demonstrated that GP knowledge surrounding complex medical issues is lacking, to the point that many GPs do not accept ME/CFS as a condition at all.^42^ Health care is becoming increasingly complex and treatment is dictated by the evidence, which may be imprecise, conflicting,^43^ or inappropriate for certain groups in society, such as women.^37^ In complex systems, unpredictability and acceptance of the unknown is inevitable. There is an urgent need for new conceptual frameworks to replace the traditional “reduce and resolve” approaches to health care.^43^

Women who were involved in patient advocacy noted a gap in the medical research and a requirement for consumer engagement. Consumer engagement is becoming increasingly acknowledged as an essential part of the health research process.^44^ Critical stakeholders such as patients and patient advocacy groups can help identify current gaps in health research and aid in consideration of the role of trauma in lived experience.^45^

The women in this study reported poor physical health and quality of life, with significant emotional and social impacts. Women with chronic conditions report poorer physical health than men with chronic conditions.^46^ Many women described daily pain, fatigue, and a significant decrease in their quality of life. This is in keeping with the literature on chronic pain, with a recent mixed-methods study of both female and male chronic pain patients showing significantly lower mean quality-of-life scores compared with the general population and patients with other long-term conditions.^47^ Respondents in this mixed methods described the adverse impacts of pain on their physical functioning, professional lives, social lives, relationships, sleep, and mental health.^47^ Similarly, a 2018 cross-sectional study of chronic pain in female breast cancer survivors demonstrated an association between pain and poorer quality of life, particularly in younger women.^48^ Another 2018 cross-sectional analysis of Australian women with chronic pain drew an association between women's response to pain, such as catastrophizing, and impact of pain on quality of life.^49^

In contrast to this cross-sectional analysis, several women in our analysis reported feeling anxiety because of their symptoms rather than the cause of them, however, there was no sense of catastrophizing, but rather a wide sense of acceptance and strength, alongside frustration at feeling dismissed. This sense of acceptance and illness as a part of life has been observed in an interview study examining the experiences of aging women with chronic conditions.^50^

Many of women interviewed described living with disabling fatigue. Fatigue is a common but poorly understood symptom of many chronic conditions^51,52^ and other studies, in keeping with our findings, have demonstrated that it is strongly associated with decreased participation in social activities.^53^ Fatigue is frequently ignored in cases where there are no laboratory findings to support a cause and unexplained fatigue is associated with a poorer quality of life.^54^ As several women in our interviews were diagnosed with conditions still considered to be medically unexplained, despite increasing evidence to the contrary, such as ME/CFS, it is possible that receiving less treatment is associated with increased fatigue and greater impacts on quality of life. Additionally, other research notes the importance of validating the experiences of women who suffer with fatigue.^55^

This study presents the experiences of Australian women with complex chronic health conditions in the health care system. In some ways, this study builds upon existing knowledge of sex and gender gaps in medical care, however, this study strongly highlights some strikingly similar adverse experiences reported by female patients. There is little prior research on female patients' experiences of medical gaslighting, dismissal, and disbelief. The work presented in this study has important implications for female patient care in an androcentric medical system. It is vital that clinicians understand and listen to their patients to provide the best and safest clinical care.

## Limitations

This research has several limitations. First, the principal researcher did not consult with patient advocates in the design of this study, however, this was mitigated to some extent by review of the article prepublication by consumer stakeholders. Women who did not speak English were unable to participate owing to lack of funding for translators, therefore, an important population group were excluded and this may impact upon transferability. Recruitment for this study occurred predominantly through social media and this may have resulted in a bias toward women who are more educated, more technologically connected and more likely to be willing to share any adverse experiences.

Many of the women in this study had MUS conditions, which is potentially a result of the lack of research highlighted; this raises questions about the transferability of findings to women with chronic conditions that have established diagnostic tests and are easier to diagnose. However, by interviewing 20 women with a range of conditions, we demonstrate that across a spectrum of conditions there are common themes in the health care experiences of Australian women with complex chronic conditions.

Finally, as with all qualitative research studies, there is the potential for researcher bias and interpretation. We have sought to mitigate this as far as possible; all data were collected and analyzed by the principal researcher, and during both processes, the researcher engaged in reflexivity as recommended by Braun and Clarke.^56^

## Conclusions

Women with complex chronic conditions in Australia report pain, fatigue, and suffering that significantly impact upon their daily lives. Despite their sense of acceptance, many women felt frustration toward medical practitioners and there was a shared feeling that the pain and suffering of women is dismissed or not taken seriously. Many women expressed trauma because of their experiences in health care and this often seemed to lead to a fear of accessing health services, even in potentially life-threatening situations. All the women interviewed highlighted a need for more knowledge, understanding, and empathy from health care practitioners surrounding the care of women with complex chronic conditions.

## Recommendations

We would recommend the following to address the adverse experiences of women in the Australian health care system:

- Addressing the research gender gap by journal requirements for researchers to recruit an appropriate number of women to clinical research and to analyze results by sex and gender
- Inclusion of women's chronic conditions in medical school curricula
- Educational sessions for clinicians and students on women's health, medical complexity, and management of chronic disease in women
- Empathy training for clinicians surrounding women's health, medical complexity, and management of medical uncertainty

## Abbreviations Used

CIN: cervical intraepithelial neoplasia
EDS: Ehlers–Danlos syndrome
GP: general practitioner
IBS: irritable bowel syndrome
IQR: interquartile range
ME/CFS: myalgic encephalomyelitis/chronic fatigue syndrome
MUS: medically unexplained symptoms
NDIS: National Disability Insurance Scheme (Australian)
POTS: postural orthostatic tachycardia syndrome
PTSD: posttraumatic stress disorder
